# The Weiss Functional Impairment Rating Scale-Parent Form for assessing ADHD: evaluating diagnostic accuracy and determining optimal thresholds using ROC analysis

**DOI:** 10.1007/s11136-017-1514-8

**Published:** 2017-02-20

**Authors:** Trevor Thompson, Andrew Lloyd, Alain Joseph, Margaret Weiss

**Affiliations:** 1 Faculty of Education and Health, University of Greenwich, London, SE9 2UG UK; 2 Bladon Associates Ltd., 3 Kings Meadow, Oxford, OX2 0DP UK; 3 Shire, Zählerweg 10, 6301 Zug, Switzerland; 4 Department of Psychiatry, Faculty of Medicine, University of British Columbia, 2255 Wesbrook Mall, Vancouver, BC V6T 1Z4 Canada

**Keywords:** Attention-deficit/hyperactivity disorder, Weiss functional impairment rating scale, Disease severity, Disease classification, Receiver operating characteristics analysis

## Abstract

**Purpose:**

The Weiss Functional Impairment Rating Scale-Parent Form (WFIRS-P) is a 50-item scale that assesses functional impairment on six clinically relevant domains typically affected in attention-deficit/hyperactivity disorder (ADHD). As functional impairment is central to ADHD, the WFIRS-P offers potential as a tool for assessing functional impairment in ADHD. These analyses were designed to examine the overall performance of WFIRS-P in differentiating ADHD and non-ADHD cases using receiver operating characteristics (ROC) analysis. This is the first attempt to empirically determine the level of functional impairment that differentiates ADHD children from normal controls.

**Methods:**

This observational study comprised 5–19-year-olds with physician-diagnosed ADHD (*n* = 476) and non-ADHD controls (*n* = 202). ROC analysis evaluated the ability of WFIRS-P to discriminate between ADHD and non-ADHD, and identified a WFIRS-P cut-off score that optimises correct classification. Data were analysed for the complete sample, for males versus females and for participants in two age groups (5–12 versus 13–19 years).

**Results:**

Area under the curve (AUC) was 0.91 (95% confidence interval 0.88–0.93) for the overall WFIRS-P score, suggesting highly accurate classification of ADHD distinct from non-ADHD. Sensitivity (0.83) and specificity (0.85) were maximal for a mean overall WFIRS-P score of 0.65, suggesting that this is an appropriate threshold for differentiation. DeLong’s test found no significant differences in AUCs for males versus females or 5–12 versus 13–19 years, suggesting that WFIRS-P is an accurate classifier of ADHD across gender and age.

**Conclusions:**

When assessing function, WFIRS-P appears to provide a simple and effective basis for differentiating between individuals with/without ADHD in terms of functional impairment.

**Classification:**

Disease-specific applications of QOL research.

## Introduction

Attention-deficit/hyperactivity disorder (ADHD) is a common neurobehavioural disorder in children and adolescents, characterised by inattention, motor hyperactivity, and impulsivity [1, 2]. Accurate diagnosis of ADHD can be challenging, as behavioural symptoms can alter in different environments and as an individual matures through adolescence [3, 4]. In addition, these symptoms are not unique and may overlap or mimic those of other disorders and this may impede successful diagnostic differentiation [2]. Furthermore, 50–90% of children with ADHD have at least one comorbid condition, with approximately half suffering from two or more comorbidities [5]. Despite the most common ADHD comorbidities being highly consistent across numerous studies, they are known to contribute to unsuccessful diagnosis of the disorder [6].

Understanding the full impact of ADHD should in part be based on the severity of any functional impairment. Therefore, measurement of the impact of ADHD should be based on the accurate evaluation and recording of symptoms, and functional impairment, using valid, reliable, and sensitive rating scales [2]. Such scales are also necessary for the evaluation of functional impairment when evaluating the efficacy of ADHD treatments [7]. This is the opinion held by the European Medicines Agency, who specify that functional outcomes should be used to assess efficacy in any ADHD clinical trial [8].

The Weiss Functional Impairment Rating Scale-Parent Form (WFIRS-P) is a 50-item scale designed to evaluate the extent to which an individual’s ability to function is impaired by any emotional or behavioural problems [2]. The scale is completed by parents or caregivers who rate impairment across a number of domains that are clinically relevant to ADHD (e.g., social activities and behavioural functioning). Several studies have explored the psychometric properties of the WFIRS-P and have demonstrated good internal consistency, with Cronbach’s alpha exceeding 0.7 for all domains, and good test–retest reliability shown for total and domain scores [9–11]. The WFIRS-P has also shown evidence of convergent validity, correlating with other ADHD-rating scales, such as the ADHD-Rating Scale-IV and Clinical Global Impressions–Severity, and has also exhibited sensitivity to treatment effects [11, 12]. The WFIRS-P has also been used in clinical settings, psychosocial research [12], and clinical trials of medication [13] to determine functional impairment in children with ADHD and its response to treatment. These studies have shown that the correlation of symptoms to functional outcome is moderate [14, 15], indicating the need to assess both symptoms and functional outcome to determine actual response to treatment. Clinical trials with stimulant medication and a summer treatment programme have also shown that functioning improves with treatment, with a previous study demonstrating that a change score of 0.25 on the WFIRS-P represents a minimally important difference of functional improvement [16]. However, few, if any, studies have examined the sensitivity and specificity of the WFIRS-P scale and thus explored its potential as a useful preliminary screening aid for possible ADHD.

The objectives of this study were, therefore, to examine the overall performance of the WFIRS-P in differentiating ADHD and non-ADHD cases using receiver operating characteristics (ROC) analysis, and identify a test score that optimises the correct classification of cases. The performance of the WFIRS-P was evaluated for both the overall and the individual domain scores, and compared across male and female subjects and those of different ages, to determine whether diagnostic accuracy was maintained across gender and age. Establishment of a WFIRS-P cut-off score that differentiates normal from abnormal functioning may also be a useful first step in establishing a rigorous empirical definition of functional impairment. Further research might then also establish empirical definitions for acceptable functioning in ADHD.

## Methods

### Study design and participants

This was an observational study comprising a sample of parents of individuals aged 5–19 years who reported that they had been diagnosed with ADHD, and a similar group of parents of non-ADHD controls who were healthy young people of the same age as the ADHD sample. Participants were recruited from the following countries: UK (138), US (128), Germany (140), Spain (64), Canada (69), France (39), Italy (50), and Netherlands (50). No formal sample size estimation was conducted, and these represent convenience samples. Recruitment specialists identified potential participants through advertising, patient advocacy groups and through treating physicians. All data in the study were collected from parents of individuals with ADHD, and it was the parents who consented to take part in the study. This study was approved by an Independent Review Board [Essex Institutional Review Board (A2190)]. No additional review was sought in each country, because this was a non-interventional survey of parents of young people with ADHD—rather than the actual patients themselves. The agency that we worked with for participant recruitment reviewed the study and agreed that it met their professional standards for survey research. All participants provided informed consent.

### Procedures

The data were collected by a specialist recruitment agency who identified the parents of children with ADHD. The data were collected through a postal survey. Parents received study packs through the post and were asked to complete and return them. Participants who had agreed to take part were followed up if they had not returned the forms. The study packs included the WFIRS-P along with demographic forms. Each individual’s ADHD status was recorded based on self-report by the parent or caregiver. The control group was recruited to provide a comparison group broadly matched for age.

The WFIRS-P is a 50-item scale that requires parents to rate the impact of their child’s emotional or behavioural problems in the previous month on six separate domains: (A) Family (ten items); (B) School and learning (ten items); (C) Life skills (ten items); (D) Child’s self-concept (three items); (E) Social activities (seven items); and (F) Risky activities (ten items). Each item is rated on a four-point scale from 0 (‘never or not at all’) to 3 (‘very often or very much’) or rated as ‘not applicable’. The mean of all scored items for each domain was computed. Each domain score, therefore, has a minimum–maximum range of 0–3. A single overall WFIRS-P score was also calculated as the average of the six domain scores.

### Analysis

ROC curve analysis provides a means to evaluate an instrument’s ability to successfully differentiate cases from non-cases for a variety of cut-off scores. In the case of the WFIRS-P, a threshold score can be applied, with scores above this threshold used to classify cases and scores below this threshold used to classify non-cases. ROC analysis allows a comprehensive evaluation of the overall discriminatory performance of the test by plotting sensitivity and specificity rates for the entire range of scores, thus enabling an optimal cut-off score that represents the highest overall level of sensitivity and specificity to be identified. In this study, non-parametric ROC was used due to a slight floor effect (positive skew) in WFIRS-P scores observed for controls. Overall WFIRS-P test scores were analysed, along with each scale domain score. Data were analysed for the complete sample, as well as for males compared with females and for participants in two age groups (5–12 versus 13–19 years).

An area under the curve (AUC) statistic was calculated to provide a single summary measure of test performance using the non-parametric trapezoidal method. Higher AUC values indicate better overall test sensitivity and specificity, with 1.0 representing perfect discrimination of cases and non-cases, and 0.5 representing chance classification. DeLong’s test for paired ROC curves was used to compare AUC values across WFIRS-P domains, with DeLong’s test for independent groups used to compare AUC values across gender and age group subsamples [17].

An optimal WFIRS-P threshold score for case differentiation was derived using Youden’s Index, a commonly used statistic that reflects overall test performance, with higher Youden’s *J* values indicating greater combined sensitivity and specificity [18], with both sensitivity and specificity given equal weighting. Therefore, the WFIRS-P score that produces the highest *J* value can be used as an optimal overall classification threshold for the study sample. All analyses were conducted using the statistical package R version 3.1.2 [19].

## Results

### Participant demographics

The participant demographics and WFIRS-P scores are presented in Table 1. A total of 678 participants were recruited, of whom 476 were ADHD cases and 202 were non-ADHD controls. The age range of the overall sample was 5–19 years with a mean of 11.46 (standard deviation 3.36) years, and consisted of 483 males and 195 females. There were more males than females in the ADHD case group, but this gender ratio is consistent with population prevalence estimates [20]. As expected, WFIRS-P overall and domain scores were higher in the ADHD case group.

**Table 1:** Participant demographics

	Controls (*n* = 202)	ADHD cases (*n* = 476)
Gender (male), *n* (%)	104 (51)	379 (80)
Age		
Years, mean (SD)	11.5 (3.4)	11.4 (3.4)
Children (5–12 years), *n*	115	278
Adolescents (13–19 years), *n*	87	198
WFIRS-P scores, mean (SD)		
Overall	0.37 (0.34)	1.18 (0.55)
A. Family	0.39 (0.42)	1.35 (0.72)
B. School and learning	0.28 (0.40)	1.21 (0.67)
C. Life skills	0.52 (0.41)	1.33 (0.63)
D. Child’s self-concept	0.33 (0.49)	1.26 (0.90)
E. Social activities	0.41 (0.45)	1.23 (0.74)
F. Risky activities	0.19 (0.30)	0.70 (0.54)

*SD* standard deviation, *WFIRS-P* Weiss Functional Impairment Rating Scale-Parent Form

### Performance of WFIRS-P

**Fig. 1:**
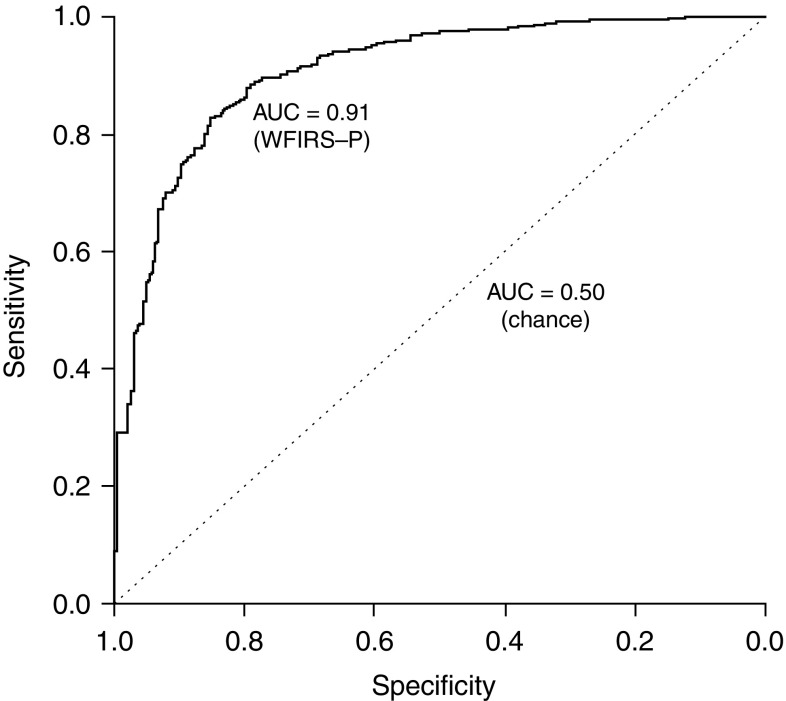
ROC curve describing differentiation of ADHD/non-ADHD across WFIRS-P total scores. *AUC* area under the curve, *ROC* receiver operating characteristics, *WFIRS-P* Weiss Functional Impairment Rating Scale-Parent Form

Sensitivity and specificity values were calculated across the entire range of overall WFIRS-P total scores and are displayed in Fig. 1. The ROC curve represents the overall performance of the WFIRS-P in successfully differentiating ADHD cases from non-ADHD controls, while the diagonal line represents chance classification (i.e., an AUC of 0.50 and that expected by a random categorization). The greater the deviation of the ROC curve from the diagonal line, the greater the successful classification performance of the scale. The deviation of the curve shown suggests that the WFIRS-P total score is highly effective in differentiating ADHD from non-ADHD cases in the study sample. The computed AUC value was 0.91 (95% confidence interval 0.88–0.93), indicating outstanding discrimination (>0.9) according to Hosmer–Lemeshow guidelines [21] and significantly higher than that expected by chance.

### Optimal threshold for WFIRS-P scores

**Fig. 2:**
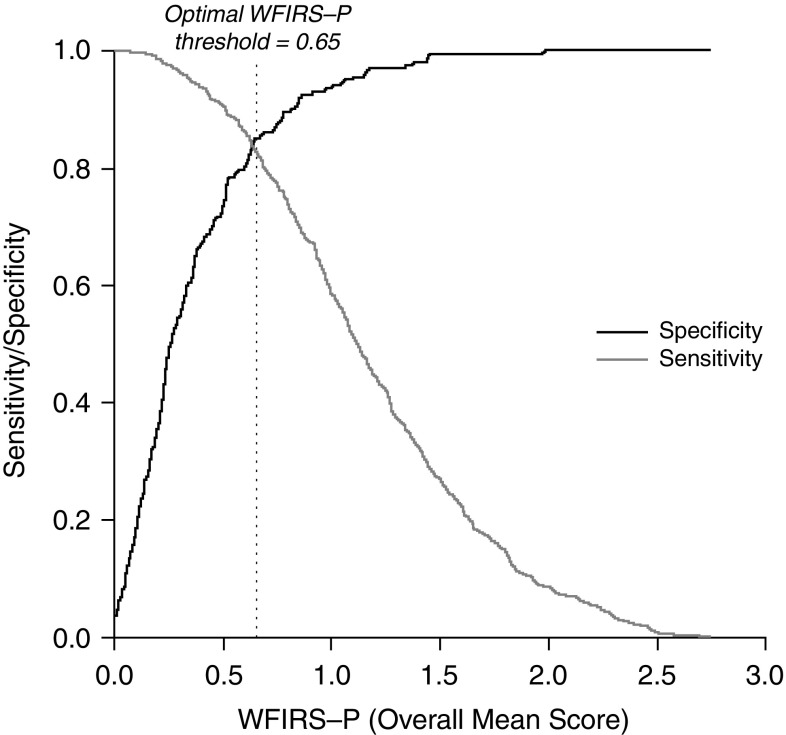
Sensitivity and specificity across the range of overall WFIRS-P total scores. *WFIRS-P* Weiss Functional Impairment Rating Scale-Parent Form

To identify an overall WFIRS-P cut-off score associated with maximum overall sensitivity and specificity, Youden’s *J* was calculated for every test score. A *J* value of 0 indicates poor overall sensitivity and specificity (e.g., 50% sensitivity and 50% specificity) for a specific cut-off score, whereas a value of 1 indicates 100% sensitivity and specificity. The computed *J* was highest (*J* = 0.68) for the WFIRS-P cut-off value of 0.65, indicating this to be the optimal cut-off value for maximising discrimination of ADHD and non-ADHD cases (Fig. 2). Categorization of individual cases with WFIRS-P scores ≥0.65 as ‘ADHD’ and <0.65 scores as ‘non-ADHD’ resulted in classification with 83% sensitivity and 85% specificity.

To examine the classification performance of individual WFIRS-P domains, ROC analysis was repeated for the six subscales. The AUCs and confidence intervals for the ROC curves of all six domains, along with optimal thresholds and associated sensitivity and specificity, are shown in Table 2. AUC statistics indicated excellent and statistically significant classification for all six subdomains ranging from 0.90 (Domain B. School and learning) to 0.81 (Domain D. Child’s self-concept). A post hoc statistical comparison of AUC values across domains, using DeLong’s test for paired ROC curves and applying a conservative Bonferroni correction of *α* = 0.003 to account for the number of comparisons, found A and B to exhibit significantly higher AUCs than domains D, E, and F (*z*’s = 2.85–5.26; *p*’s < 0.003). Given the apparent classification superiority of these domains, the performance of A and B domain scales was compared with the overall WFIRS-P scores. Results provided some evidence that the AUC of the overall WFIRS-P may be greater than that of domains A (*p* = 0.002) and B (*p* = 0.052). However, the difference in actual AUC values between the overall total scale (0.91) and domains A (0.90) and B (0.88) is minimal and suggests that overall performance is similar across scales in terms of clinical relevance.

**Table 2:** AUC and optimal cut-off statistics for WFIRS-P domains

WFIRS-P domain	ROC curves			Optimal threshold scores		
	AUC	95% CI	Scale score	*J*	Sensitivity	Specificity
A. Family	0.88	0.87–0.90	0.75	0.63	0.84	0.78
B. School and learning	0.90	0.88–0.91	0.70	0.64	0.86	0.78
C. Life skills	0.86	0.84–0.88	0.78	0.56	0.75	0.81
D. Child’s self-concept	0.81	0.80–0.83	1.00	0.51	0.85	0.66
E. Social activities	0.84	0.82–0.85	0.71	0.54	0.79	0.75
F. Risky activities	0.85	0.83–0.86	0.22	0.56	0.75	0.81
Overall total score	0.91	0.88–0.93	0.65	0.68	0.83	0.85

*AUC* area under the curve, *CI* confidence interval, *J* Youden’s *J* value, *ROC* receiver operating characteristics, *WFIRS-P* Weiss Functional Impairment Rating Scale-Parent Form

### Results by gender and age

**Fig. 3:**
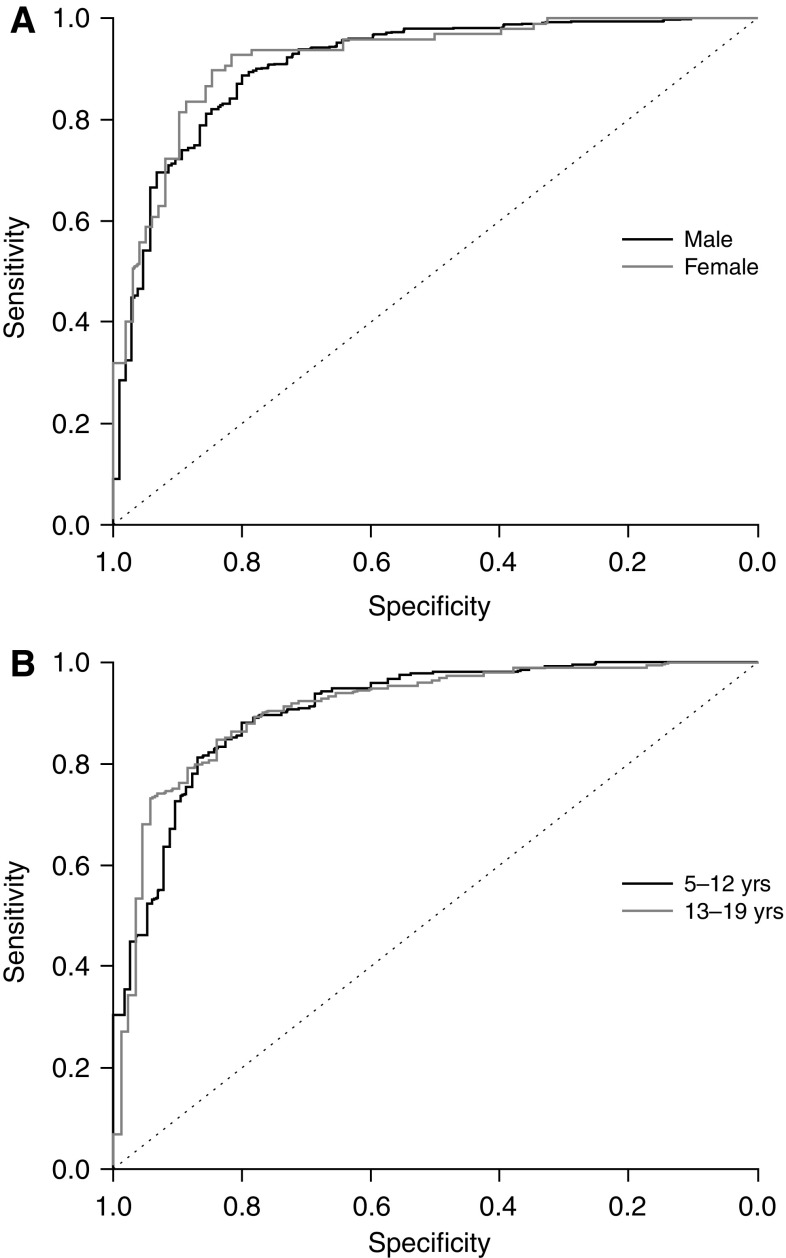
ROC curves for the overall WFIRS-P total score across gender (**a**) and age group (**b**). *ROC* receiver operating characteristics, *WFIRS-P* Weiss Functional Impairment Rating Scale-Parent Form

ROC curves depicting the sensitivity and specificity of the overall WFIRS-P scores for males and females are shown in Fig. 3a. A statistical comparison of AUCs for males versus females using DeLong’s test for independent ROCs revealed no significant gender differences in AUC for either the overall or domain scales (*D* = 0.09–1.58; *p* = 0.93–0.11). Similarly, ROC analysis and DeLong’s test results comparing WFIRS-P scores for participants aged 5–12 years with those aged 13–19 years revealed no significant differences for either the overall or domain scales (*D* = 0.36–0.99; *p* = 0.72–0.33; Fig. 3b).

## Discussion

The primary aim of this analysis was to evaluate the ability of WFIRS-P to accurately differentiate functional impairment in individuals with ADHD from broadly age-matched controls and to determine an optimal classification cut-off score for defining functional impairment. Results indicated that the WFIRS-P overall total score and domain scores did provide accurate classification and differentiation of functional impairment in those with and without ADHD. Although the overall scale and the Family (A) and School and learning (B) domains were associated with especially high levels of overall classification, differences between domain scores were minimal and unlikely to be clinically important. Although it would be injudicious to consider a cut-off value from a single statistical index as a definitive threshold, results from the current study provide guidance for consideration of a generalised WFIRS-P cut-off value of around 0.65 for which sensitivity and specificity are likely to be high. Although the AUC indicated an ‘outstanding’ classification according to the standard guidelines [21], it would be unwise to interpret this as indicating that the WFIRS-P should be used as a diagnostic assessment. Rather, the scale may have utility as a quick and easily administered preliminary screener if ADHD is suspected, but further clinical evaluation would be essential both to confirm diagnosis of ADHD and to investigate other comorbidities or disorders. Figure 2 can be used as a guide to the approximate sensitivity or specificity values that might be expected for any overall WFIRS-P cut-off score. An instrument with an optimised score can help to maximise accuracy and precision in defining functional impairment and a paradigm for functional improvement and remission. The findings also indicate that the WFIRS-P appeared to be an equally effective classifier and with similar optimal threshold values for males and females, and for children (5–12 years) and adolescents (13–19 years).

Several limitations of the study should be noted. First, the study sample contained comparatively fewer females than males. Although the total number of females in both ADHD and control groups was adequate for analysis, replication of the current results (especially those relating to gender) is ideally required before extrapolating to the wider population. In addition, the optimal classification cut-off score we obtained here for children may be different for adults when a self-report version of the scale is used or, given that diagnostic criteria may vary slightly across regions when used in a different country. As such, future studies could examine the impact of these factors on both the overall classification performance and the identification of an appropriate cut-off score. Third, although the WFIRS-P did demonstrate accurate discrimination of functional impairment between ADHD and non-ADHD, it may be that alternative scoring methods may provide even better classification. For example, the mean value for the scale may sometimes fail to capture functional impairment that is driven by a few salient and severely impaired items (e.g., a child may be doing relatively well in many areas, but one or two items are causing severe difficulty), and this could suggest an alternative scoring procedure that accounts for this and could offer superior performance. For example, apart from overall mean score, children with either one symptom that is severe (3) or two symptoms that are moderate (2) within a domain could be considered functionally impaired in that domain. DSM-IV diagnosis of ADHD required that a child shows functional impairment in at least two settings or domains. The DSM-5 Disability Group recommend use of the WHODAS 2.0 which is based on the International Classification of Functioning, Disability and Health [22]. The WHODAS 2.0 is a self-report, appropriate for adults but not for children. The WHODAS is not specific to the impact of ADHD, and is not appropriate for use with children and has not been widely adopted [23]. The WFIRS-P is an alternative that allows for an empirical measure to further define the DSM-5 requirement of disability for diagnosis, based on an empirical measure in widespread use, that is well validated, designed for measurement of functional impairment secondary to ADHD, and to be completed by parents.

The control group was recruited with the same age range as the ADHD group, which meant that the two groups were broadly matched in terms of mean age but were not matched on an individual basis. A key limitation of this study is that verification of diagnosis (e.g., ADHD-Rating Scale-IV scores) were not available, which meant that we relied on self-reported diagnosis of ADHD. Although we have no strong reason to believe that this would introduce any systematic bias, this could potentially affect the precision of the sensitivity and specificity estimates of the scale in differentiating between cases and controls.

In this study data set, detailed information regarding disease severity was not available, so it was not possible to consider this in any analysis. Finally, and importantly, while the WFIRS-P appeared to accurately discriminate functional impairment between ADHD and non-ADHD controls, further work would be needed to examine the ability of the WFIRS-P to differentiate how and to what extent the functional impairment identified on this measure is specific to ADHD.

## Conclusion

This study empirically examines the threshold that differentiates functional impairment in ADHD versus non-ADHD individuals. Current practice guidelines and diagnostic criteria have established that diagnosis and measurement of outcome must address both symptom criteria and functional impairment. We have good symptom rating scales and empirically based cut-off scores that define a generally agreed upon cut-off score for symptoms. This study has established that a mean score of 0.65 on the WFIRS-P reliably differentiates individuals with ADHD who are functionally impaired from controls. As well as confirming that ADHD is associated with considerable functional impairment compared with normal controls across several domains, the current findings also endorse the ability of the WFIRS-P to provide accurate classification of functional impairment in ADHD across gender and for both children and adolescents, and provide a suggested general cut-off score to help optimise classification. These results support the potential for the WFIRS-P as a quick and easily administered assessment of functional impairment in ADHD, which could be used to prompt further, more detailed, clinical investigation.
